# Full‐length 16S rRNA gene classification of Atlantic salmon bacteria and effects of using different 16S variable regions on community structure analysis

**DOI:** 10.1002/mbo3.898

**Published:** 2019-07-04

**Authors:** Terje Klemetsen, Nils Peder Willassen, Christian René Karlsen

**Affiliations:** ^1^ Department of Chemistry, Center for Bioinformatics UiT The Arctic University of Norway Tromsø Norway; ^2^ Department of fish health Nofima Norway

**Keywords:** Atlantic salmon, Full‐length 16S rRNA gene sequence, microbiota

## Abstract

Understanding fish‐microbial relationships may be of great value for fish producers as fish growth, development and welfare are influenced by the microbial community associated with the rearing systems and fish surfaces. Accurate methods to generate and analyze these microbial communities would be an important tool to help improve understanding of microbial effects in the industry. In this study, we performed taxonomic classification and determination of operational taxonomic units on Atlantic salmon microbiota by taking advantage of full‐length 16S rRNA gene sequences. Skin mucus was dominated by the genera *Flavobacterium* and *Psychrobacter*. Intestinal samples were dominated by the genera *Carnobacterium, Aeromonas, Mycoplasma* and by sequences assigned to the order Clostridiales. Applying Sanger sequencing on the full‐length bacterial 16S rRNA gene from the pool of 46 isolates obtained in this study showed a clear assignment of the PacBio full‐length bacterial 16S rRNA gene sequences down to the genus level. One of the bottlenecks in comparing microbial profiles is that different studies use different 16S rRNA gene regions. Comparisons of sequence assignments between full‐length and in silico derived variable 16S rRNA gene regions showed different microbial profiles with variable effects between phylogenetic groups and taxonomic ranks.

## Graphical abstract

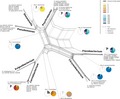

## 1 BACKGROUND

Second‐generation sequencing is widely used to assess the composition of the microbial community through partial sequence analysis of the 16S rRNA gene. Different bacterial 16S rRNA gene regions are used by different researchers, which makes it difficult to perform global comparisons of microbiome studies. Discrepancy in bacterial diversity may also be observed between full‐length 16S rRNA gene and variable region 16S rRNA gene datasets (Sun, Jiang, Wu, & Zhou, 2013; Wagner et al., 2016). Sequence regions but also PCR primer choice used for short‐read amplicon sequencing of different 16S rRNA gene hypervariable regions can affect the accuracy of the inferred community profiles and sensitivity to certain bacterial taxa (Chen et al., 2019; Walker et al., 2015). Therefore, sequences of full‐length 16S rRNA genes that cover all the variable regions should potentially increase the accuracy and the resolution of closely related taxa. However, full‐length sequences compared to shorter sequences generated by other platforms favorized Proteobacteria and provided a lower taxonomic profiling of the human feces, partly due to sequence accuracy and low coverage of terminal regions in the 16S rRNA databases (Whon et al., 2018). Still, long‐read sequencing such as PacBio circular consensus sequencing (CCS) applied on the 16S rRNA gene provides a promising approach to increase the taxonomic resolution of microbial communities. The CCS technology generates a consensus sequence from a single molecule by reading a ligated circular DNA template multiple times, achieving high read accuracy (Travers, Chin, Rank, Eid, & Turner, 2010). However, few studies have investigated advantages and disadvantages of PacBio CCS for such analyses. A recent study showed that PacBio sequencing error rates were in the same range as Roche 454 and MiSeq platforms (Wagner et al., 2016). The authors reported inconsistencies in species‐level analysis between full‐length 16S rRNA gene sequences obtained from Sanger and PacBio sequencing comparisons and that more sample types were needed to determine whether partial or full‐length 16S rRNA gene sequences was superior in terms of taxonomic profiling effectiveness. The first metagenomic marine environmental samples from PacBio CCS sequences provided a superior taxonomic resolution to the species level compared to in silico derived partial regions of the gene (Pootakham et al., 2017).

In fish, both the skin epithelial surface and the gastrointestinal tract are covered by a mucosal layer. The main components of this mucus layer are secreted glycoproteins called mucins, which are differentially regulated between tissue types in Atlantic salmon, *Salmo salar* (Sveen, Grammes, Ytteborg, Takle, & Jørgensen, 2017). These glycosylated proteins might be a highly attractive substrate for the attachment and settlement of microorganisms. Interaction studies between cutaneous microbiota and the fish surface are scarce, but mutualistic relationships (Beklioglu, Telli, & Gozen, 2006) and the existence of a resilient microbiome (Larsen, Bullard, Womble, & Arias, 2015) has been suggested. Salinity acclimation results in turnover of dominant bacterial taxa within the host microbiome that are unrelated to changes in water microbiota (Schmidt, Smith, Melvin, & Amaral‐Zettler, 2015), which is also reported for Atlantic salmon (Karlsen et al., 2017; Lokesh & Kiron, 2016). Microorganisms populating the gastrointestinal tract are believed to take part in digestive functions and contribute to fish health (Nayak, 2010). Many fish gut bacteria are not detected from water samples (Sullam et al., 2012), and gut microbiota profiles in Atlantic salmon change between intestinal compartments (Gajardo, Rodiles, et al., 2016), rearing environments (Dehler, Secombes, & Martin, 2017), and diets (Schmidt, Amaral‐Zettler, Davidson, Summerfelt, & Good, 2016). Sequencing the 16S rRNA genes is a powerful tool that provides a comprehensive picture of the phylogenetic diversity and composition of the microorganisms present in a sample as many of the microbial groups are absent or difficult to cultivate. Traditionally, diagnosis of suspected Atlantic salmon bacterial infections has relied on clinical signs and symptoms, and microbiological culture‐dependent methods. Many bacteria associated with salmonids have been considered difficult to cultivate and more accurate culture‐independent diagnostic procedures are developed (Grove, Reitan, Lunder, & Colquhoun, 2008; Sepúlveda, Bohle, Labra, Grothusen, & Marshall, 2013). However, culturing is an important step to better understand effects of microorganisms. Recovering isolates of microbial symbionts is increasing in focus as they can be used to study activity and functional relationships to a host (Esteves, Amer, Nguyen, & Thomas, 2016; KleinJan, Jeanthon, Boyen, & Dittami, 2017). The present study was also designed to recover and identify members of the Atlantic salmon microbial communities, characterized by 16S rRNA gene sequencing, for future studies.

The changing environment in the salmon production, utilization of different feeds, use of closed or semi‐closed recirculation systems and transfer to unprotected seawater environment at the final stage of production affects skin and gut of complex microbial communities. Taxonomic profiling that can reveal alterations or deviations from “normal” microbial communities may advance our understanding of any functional effects of detected microbiota. Appropriate methods that accurately generate and analyze the microbial communities would be an important tool to help improve understanding of microbial effects in the aquaculture industry. Here, we applied long‐read technology to demonstrate its utility, as a proof of concept, in characterizing the microbial profiles of the skin surface and the bulk intestinal content of Atlantic salmon.

## 2 MATERIALS AND METHODS

### 2.1 Sample collection and DNA extraction

This study utilized fish from an industry research study conducted at 12 ppt salinity with recirculated water with temperature of 13°C at the Research Station for Sustainable Aquaculture (Sunndalsøra, Norway) in accordance with regulations of the Norwegian Food Safety Authority. As proof of concept, six Atlantic salmon were selected for sampling, anesthetized with benzocaine and killed by a blow to the head. The six sampled individuals were split between two dietary groups. Three had been fed fish meal (mean ± *SEM* body weight was 193 ± 41 g), and three were from a diet where fish meal was substituted with krill meal (mean ± *SEM* body weight was 181 ± 20 g). Fish were not fed for 48 hr prior to sampling. Skin mucus samples were obtained by swiping a cell swiper across the left side of the fish and concentrating mucus, which was aspirated by pipetting. The abdominal cavity was then opened and an incision was made to open the distal intestine. The intestinal content was collected by gently scraping the intestine to collect bulk feces including the intestinal mucus layer. All tissue material was stored in 96% EtOH. DNA was extracted from 200 mg skin mucus samples and 100 mg intestinal samples. The protocol was performed using the PowerLyzer® PowerSoil® DNA Isolation Kit (MoBio) according to the manufacturer's specification with the following amendments: samples after adding Solution C1 were heated at 70°C for 10 min. Samples were homogenized with the mechanical bead beater device Precellys®24 (Bertin Technologies) for 1 × 20 s at 5,000 rpm. The DNA was re‐suspended in 30 μl of DNase/RNase free molecular water and concentration determined using a Thermo Scientific Nanodrop 2000c.

### 2.2 Bacterial isolation and identification

Bacteriology was performed on the same sampled individuals for the total bacterial DNA sequence analysis. Skin mucus (200 mg) and bulk intestinal feces/mucus (100 mg) were separately vortexed and suspended in total of 1 ml 0.9% NaCl saline solution. In addition, gill mucus material and water were similarly suspended in saline solution to retrieve bacterial isolates, included in the Appendix A. A 100 μl aliquot of the content was serial diluted up to 10^–6^ and plated in duplicate onto R2A (BD Difco), representing low‐nutrient conditions and MacConkey agar (CM007 Oxoid), representing high‐nutrient conditions, under aerobic incubation at 12°C for 9 days. Plates were inspected and colony numbers were counted based on morphological characteristics, that is, pigmentation, colony form, elevation, surface appearance, and texture. The relative distribution in percentage between colony morphologies is provided as an average of each sample type. Representative colonies were selected according to dominant morphologies and then identified by 16S rRNA gene sequence analysis. Briefly, representative colonies were selected for purity plating onto R2A or MacConkey plates. Pure colonies determined for freeze stocks were further expanded in Luria‐Bertani (LB) broth (Bertani, 1951) with 3.5% NaCl at 12°C before supplementation with 10% glycerol and stored at −80°C. Genomic DNA isolation was performed using PureLink® Genomic DNA Mini Kit (Invitrogen). PCR amplification of the 16S rRNA gene with primers 27F (5′‐AGAGTTTGATCMTGGCTCAG) and 1492R (5′‐TACCTTGTTACGACTT) was identical to previous descriptions (Karlsen et al., 2014). Products were visualized following agarose (1.0%) gel electrophoresis and RedSafe (Chembio), before being purified using a QIAquick Gel Extraction Kit (Qiagen) followed by Sanger sequencing (sequenced at GATC Biotech, DNA sequencing services and bioinformatics, Germany). The forward and reverse sequences were assembled in Bioedit (Hall, 1999) and consensus sequences deposited in GenBank (submission: SUB3162162), with accession numbers MG263463‐MG263508 (Table A1, Appendix). Sequences were aligned with type strain reference sequences using Sequence Match software from The Ribosomal Database Project II (RDP II) web site (Cole et al., 2014). The phylogenetic relationships between sequences were constructed utilizing selected 16S rRNA sequences of type strains in each genus (A1). Sequences were aligned using the ClustalW algorithm in BioEdit (Hall, 1999). The phylogenetic relationships were determined using maximum likelihood (ML) based on the Tamura 3‐parameter model including all coding positions (total of 1,425) with 1,000 bootstrap trials (neighbor‐joining tree) in MEGA6 (Tamura, Stecher, Peterson, Filipski, & Kumar, 2013).

### 2.3 PCR amplification, barcoding, and PacBio sequencing

To analyze the microbial population associated with the skin mucus and intestine, sequencing of the 16S rRNA gene was performed using the PacBio sequencing technology (Pacific Biosciences). The full‐length 16S rRNA gene was amplified using degenerated versions of the universal bacterial 16S rRNA gene primers 27 F (5′‐ AGRGTTTGATYMTGGCTCAG) and 1492 R (5′‐GGYTACCTTGTTACGACTT). In accordance with “Guidelines for Using PacBio® Barcodes for SMRT® Sequencing” guide, a 5 nt (5′‐GGTAG) padding sequence was added to each unique 16 nt barcode to allow all barcodes to ligate to the SMRTbell™ adapter with equal efficiency. The utilization of barcodes allowed the multiplex sequencing of amplicons from several samples in one library using SMRT®. Primers were synthesized and HPLC‐purified as recommended in PacBio's SMRT guidelines by Invitrogen Custom DNA Oligos (Thermo Fisher Scientific). Barcoded 16S rRNA amplicons were obtained by a two‐step amplification protocol using Phusion® High‐Fidelity PCR Master Mix (Thermo Fisher Scientific). The first PCR was performed in triplicate with the 27 F and 1492 R universal bacterial 16S rRNA gene primers using 100 ng of extracted total DNA, 1 × Phusion Master Mix, 0.5 μmol/L 16S‐F forward primer, 0.5 μmol/L 16S‐R reverse primer, in a 50 μl reaction volume. Samples were prepared on ice and amplified in the thermocycler with the block preheated to 98°C. The reactions were performed using the following cycling conditions: preincubation at 98°C for 2 min, followed by 25 cycles of denaturation at 98°C for 10 s, annealing at 55°C for 15 s, elongation at 72°C for 60 s, and a final extension step at 72°C for 3 min. Triplicate samples were pooled, and amplification was verified by 1% agarose gel electrophoresis before the reaction products were purified with an Invitrogen™ PureLink™ PCR Purification Kit. The purified PCR products were diluted and triplicates of 1 ng DNA was used as template for the second amplification reactions to generate padded barcoded products, with reagent concentrations as described above. Product amplification was as above with the following changes: 14 cycles with annealing at 60°C for 15 s. Triplicates were pooled, and products were verified by agarose gel electrophoresis and padded barcoded 16S rRNA gene amplicons from the reaction were purified using PureLink™ PCR Purification Kit and quantified using a NanoDrop spectrophotometer. Purified barcoded amplicons from the 12 samples (skin and intestine from 6 fish) were then pooled in equimolar concentrations, and 250 ng of DNA was used for library preparation at the Norwegian Sequencing Centre (http://www.sequencing.uio.no). Briefly, library was prepared using PacBio 2 kb library preparation protocol. Size selection was performed using Ampure beads. Adapters were ligated onto the barcoded amplicons, and the library was sequenced on a PacBio RSII system using the P6‐C4 polymerase and chemistry with a 360‐min movie time, using one SMRT cell.

### 2.4 Sequence data analysis

Raw reads were filtered and demultiplexing using RS_subreads.1 pipeline on SMRT Portal (software version 2.3) with the following settings: minimum number of passes = 1, minimum predicted accuracy = 0.90, and minimum barcode score = 30. Read sequences were then prepared by filtering to a window length between 1,000 and 1,600 nt using PRINSEQ v.0.20.4 (Schmieder & Edwards, 2011) and reoriented in accordance with SILVA v.132 SSU (Pruesse et al., 2007) with the USEARCH v.10 (Edgar, 2010) orient function. The hypervariable regions v3‐v4 and v5‐v6 in the filtered data were extracted after using BLASTN v.2.6.0 (Altschul, Gish, Miller, Myers, & Lipman, 1990) (word size 4, gap opening penalty 0, *E*‐value 0.01) to mark the flanking regions as described elsewhere (Pootakham et al., 2017). Sequences positively identified with both v‐regions were advanced to generate two new trimmed data subsets for the v3‐v4 and v5‐v6 regions. Sequences were discarded if a flanking region could not be determined, suggesting a missing or partial v‐region for a given sequence.

LCAClassifier (Lanzén et al., 2012) was applied to obtain the taxonomic mapping of the datasets (sample acronyms I1‐I6 and SM1‐SM6) and their respective data subsets. These were individually aligned against the SilvaMod database by MEGABLAST (Morgulis et al., 2008) (identity cutoff = 75.0, E‐value cutoff = 0.001) as recommended prior to applying LCAClassifier. Finally, the analysis was carried out using default settings in the LCAClassifier program. Numerical data output from taxonomic classification was ordered based on ranks of taxonomy and the assignments obtained for the 12 datasets and data subsets. This was used to calculate the variation in taxon mapping between full‐length PacBio CCS 16S rRNA gene sequences and their respective v‐regions. To detail taxonomic variations as principal components linked to the sampling sites of intestine and skin mucus only the full‐length 16S rRNA gene sequences were considered. The class level was used due to a 98.92% or greater successful assignment of the filtered full‐length sequences of any sample. The sample dataset sizes were downscaled to sample SM2, which had the lowest number of successfully assigned sequences. The scaled values of class data, treating zero as “NA,” were uploaded to ClustViz (Metsalu & Vilo, ) with parameters set to not perform row scaling and the method set to SVD with imputation. Rarefaction analysis on the obtained operational taxonomic units (OTUs) was conducted using MicrobiomeAnalyst (Dhariwal et al., 2017). Unscaled OTU counts from mapping of reads with USEARCH were provided alongside their taxonomy lineage to genus level. Filtering of data was set using default parameters. Data were not rarefied or transformed, but total sum scaling was applied. The rarefaction curve was obtained with the filtered data using 20 steps.

Cultivability was determined using the 43,910 pooled full‐length sequences combined with the 46 plate‐isolated strains. Next, the USEARCH function cluster_fast was applied to cluster all sequences within an identity threshold of 97%. Sequences in clusters containing at least one of the cultivated strains were included when computing the cultivability percentage. OTUs were also determined for the pooled dataset of 43,910 full‐length 16S rRNA gene sequences positive for v3v4 and v5v6 regions. USEARCH was applied following dereplication of the dataset, clustering OTUs at a 97% identity threshold, keeping parameters at default. 31,634 (~72%) of the full‐length sequences were successfully reassigned to the 10 OTU representatives found and counted according to sample origin. The sum of sample specific assignments was scaled to fit the smallest sample size of SM2 and incorporated as pie charts in the phylogenetic inference described below. The OTU centroid sequences were further used to infer phylogenetic relationship between these representatives along with the 46 plate‐isolated strains and 10 reference type strains. The 66 sequences were aligned with Clustal Omega v.1.2.1 (Sievers & Higgins, 2014) using default nucleotide parameters. The complete alignment was inferred using NeighborNet network with Uncorrected P distances in SplitsTree v.4.13.1 (Huson & Bryant, 2006).

## 3 RESULTS

### 3.1 Characteristics of full‐length 16S rRNA gene sequencing

**Figure 1:**
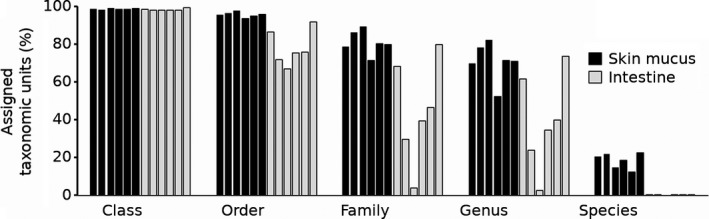
Percentage of taxonomic units assigned as reads at the class, order, family, genus, and species levels. Black bars = sampled fish skin mucus (fish no. 1 – 6). Gray bars = sampled intestine (fish no. 1 – 6)

Full‐length 16S rRNA gene sequences were amplified from DNA extracted from sampled bulk intestinal content and skin mucus of six Atlantic salmon. Amplicons were generated using a two‐step PCR approach with asymmetrical primers during the second round of amplification for a more cost‐effective way to multiplex amplicons from several samples. A total of 110,818 reads were obtained with an average read length of 23,881 nt. Of these were 76,723 assembled and demultiplexed into CCS reads with an average accuracy of 98.56% and a mean length of 1,252 nt. The average number of full passes of the CCS reads was 15.6. The number of processed full‐length 16S rRNA sequences per sample ranged from 1,483 to 7,634 reads (Table 1). The microbiota composition was determined by a phylotyping approach directly allocating sequences into taxonomic groups based on bitscore and identity threshold. Of the trimmed reads used for assignment, an average of 96.9% of the skin mucus and 78.8% of the intestine were aligned to the taxonomic level order. According to the taxonomic resolution from order to genus, this method assigned more reads to taxonomic references for skin mucus samples compared to intestinal samples (Figure 1). The discriminant taxon, based on the generated reads from the intestine samples was the prominent Clostridiales that became unassigned at higher resolution ranks. The rarefaction analysis (Figure A2) of clustered OTUs showed that the bacterial communities of the intestines are less diverse compared to the skin mucus samples. Convergence were reached (sample I4, I5, I6, SM1, and SM3) but seven out of twelve samples, including both intestine and skin mucus, did not converge properly in the analysis.

**Table 1:** Sample names, acronyms, and PacBio CCS sequence characteristics

	Sample	Acronym	PacBio CCS reads	Total assignments
Krill meal	Fish 1 Skin Mucus	SM1	4,481	3,675
	Fish 1 Intestine	I1	4,681	4,025
	Fish 2 Skin Mucus	SM2	1,483	1,226
	Fish 2 Intestine	I2	4,810	4,195
	Fish 3 Skin Mucus	SM3	2,816	2,424
	Fish 3 Intestine	I3	7,634	6,664
Fish meal	Fish 4 Skin Mucus	SM4	4,138	3,440
	Fish 4 Intestine	I4	4,971	4,300
	Fish 5 Skin Mucus	SM5	4,022	3,155
	Fish 5 Intestine	I5	5,375	4,581
	Fish 6 Skin Mucus	SM6	4,945	3,960
	Fish 6 Intestine	I6	2,633	2,265

### 3.2 Comparisons of microbial compositions

**Figure 2:**
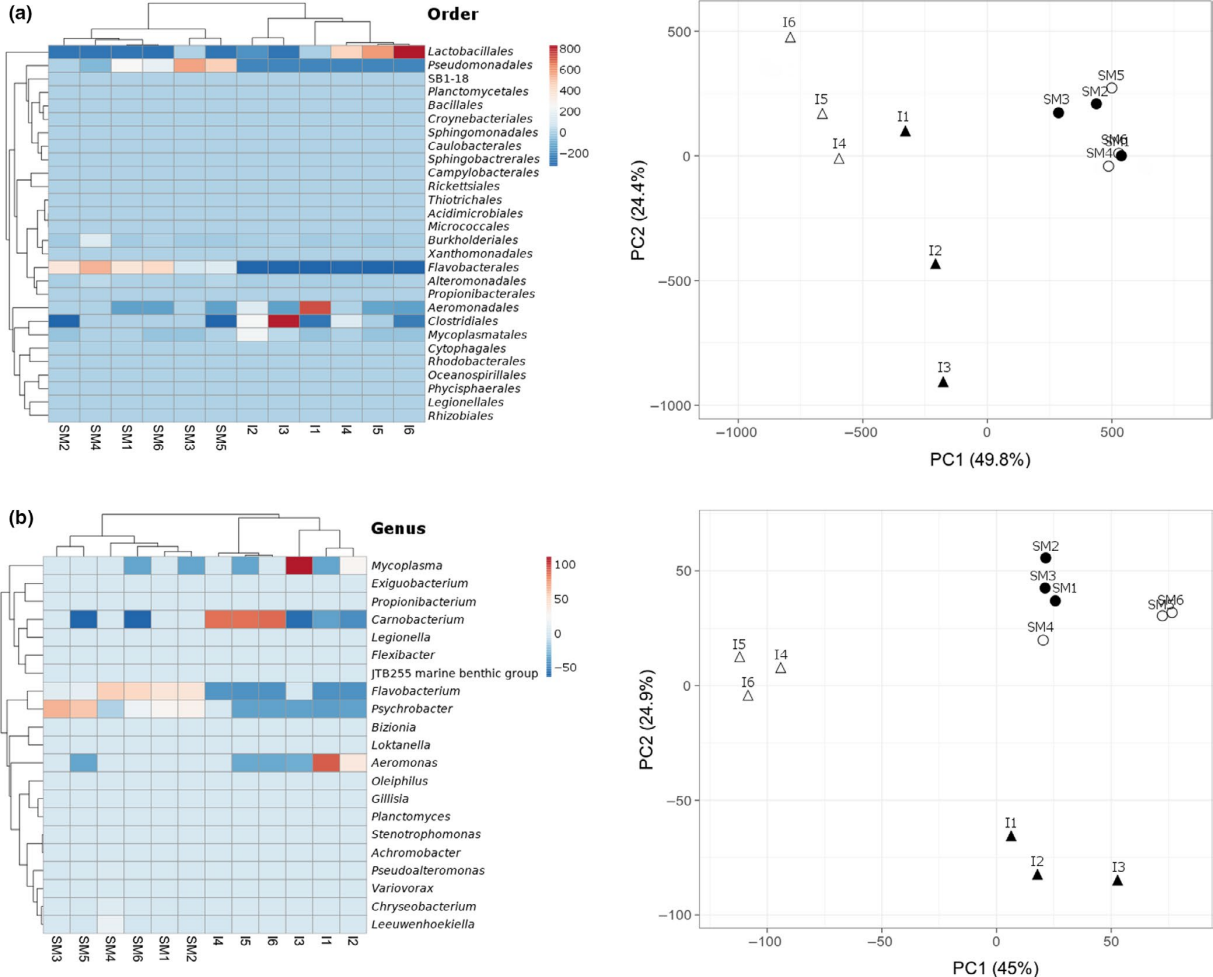
A hierarchically clustered heatmap of the microbial profiles of Atlantic skin mucus and intestine based on SILVA database taxonomy assigned to (a) order and (b) genus levels. Dendrogram at the top of the heatmap shows the clustering of the sample types, skin mucus (SM1‐SM6), and intestine (I1‐I6). The dendrogram at the left side shows the distribution of bacteria. The color scale depicts the normalized relative abundance of each rank level. Principal component analysis (PCA) plot of scaled microbiota profiles representing both skin mucus (circles) and intestinal samples (triangles) of Atlantic salmon is depicted in the far right for both order and genus levels. Filled (black) symbols for fish fed krill meal diet and unfilled (white) symbols for fish fed fish meal based diet

Hierarchical clustering strengthened by principal component analyses (Figure 2) revealed that bacteria communities clustered to tissue types sampled from the Atlantic salmon in both the taxonomic rank of order to genus. Skin mucus microbiota profiles are tightly clustered. The intestinal microbiota profiles are dispersed but separates into krill meal diet (acronym I1 – I3) with predominate *Aeromonas* and *Mycoplasma* at the genus level, and fish meal diet (acronym I4 – I6) with predominant *Carnobacterium* at the genus level. The most dominant bacteria orders of the skin mucus samples were Flavobacteriales (51.2%), Pseudomonadales (40.7%), Burkholderiales (2.3%), and Alteromonadales (1.3%). Members of the Flavobacteriales assigned to the genus level were dominated by *Flavobacterium *> *Chryseobacterium* > Leeuwenhoekiella > *Bizionia* > Gillisia. Genus members within the order of Pseudomonadales were dominated by *Psychrobacter *> *Pseudoalteromonas*. Sequences within remaining orders were not assigned down to genus level. The most dominant bacterial orders of the intestinal samples were Lactobacillales (35.0%) with *Carnobacterium* at the genus level, Clostridiales (26.1%), Aeromonadales (16.0%) with *Aeromonas* at the genus level, Mycoplasmatales (4.1%) with *Mycoplasma* at the genus level. Sequences within Clostridiales were not assigned down to the genus level.

**Figure 3:**
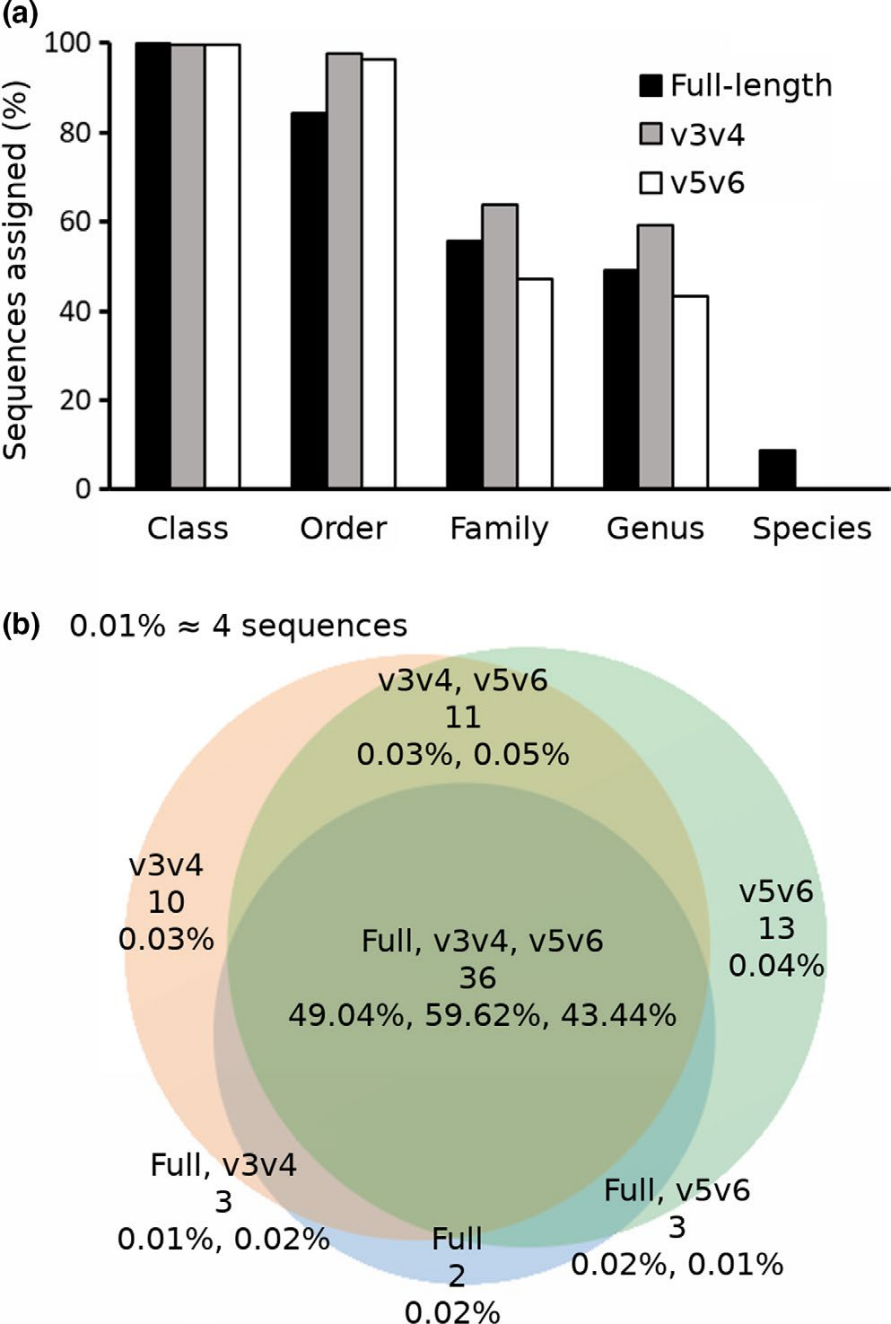
Percentage of assigned sequences at the class, order, family, genus, and species levels from full‐length (black bars), v3v4 (gray bars), and v5v6 (white bars) datasets (a). Venn diagram of genus level assignments illustrating the number of taxa shared in and between each dataset (b). Top line shows dataset(s), middle line shows the number of unique genus taxa shared among dataset(s), bottom line shows the relative abundance of sequences assigned to the involved taxa and the given dataset(s)

To determine whether longer sequences of the 16S rRNA gene would be advantageous to assign sequences to the lower ranks of taxonomic affiliation, partial sequences spanning the v3v4 and v5v6 regions were extracted in silico from the full‐length 16S rRNA gene sequence dataset. The proportion of assigned sequences between the datasets at the class, order, family, genus, and species levels were evaluated (Figure 3a). Using the SilvaMod database showed that overall sequence assignments varied between the full‐length 16S rRNA sequences and partial 16S rRNA sequences in addition to differences in the proportions of the assigned sequences at the different taxonomic ranks (Table 2). The v3v4 sequences had the highest proportion of assignment at the family and genus levels. At the species level, the full‐length dataset had the highest proportion of assigned sequences (mean 8.9%).

**Table 2:** Assignments for pooled data of full‐length 16S rRNA, v3v4 and v5v6 regions

Or.	Fa.	Ge.	Sp.	Full	v3v4	v5v6
Aeromonadales				3,242	+14	+5
Aeromonadaceae				2,801	+369	+302
*Aeromonas*				2,598	+420	+407
Alteromonadales				260	+13	+22
Pseudoalteromonadaceae				126	+103	+93
*Pseudoalteromonas*				94	+118	+117
Bacillales				34	+170	+7
Burkholderiales				591	+35	+28
Alcaligenaceae				387	+171	+142
Clostridiales				8,163	+5,264	+5,157
Ruminococcaceae				0	+11	+176
Enterobacteriales				0	0	+72
Flavobacteriales				9,391	+119	−3
Flavobacteriaceae				7,877	+1,219	+1,117
*Chryseobacterium*				154	+155	+141
*Chryseobacterium marinum*				84	−84	−84
*Flavobacterium*				6,500	+1,281	+1,058
*Flavobacterium frigidarium*				3,802	−3,802	−3,802
*Leeuwenhoekiella*				256	+235	+187
*Bizionia*				19	+19	+24
Others				13	+31	+40
Lactobacillales				7,420	−83	−217
Carnobacteriaceae				6,306	+873	−6,283
*Carnobacterium*				5,630	+1,303	−5,615
Mycoplasmatales				955	+70	+61
Mycoplasmataceae				756	+217	+201
*Mycoplasma*				571	+251	+343
Pseudomonadales				6,953	+66	+74
Moraxellaceae				6,114	+619	+523
*Psychrobacter*				5,645	+756	+790
Rhodobacterales				44	+20	+7
Rhodobacteraceae				23	+32	+19
*Sphingobacteriales*				56	+16	+10
Saprospiraceae				23	+25	+26

Note

Sum of assignments where the sub‐datasets of v‐regions are displayed relative to the full dataset. Only assignments above 0.1% (>44 no. of sequences) of the total 43,910 sequences represented in each of the three datasets is shown.

Abbreviations: Fa., family; Ge., Genus; Or., order; Sp., Species.

Figure 3b gives insight into the distribution of genus diversity in the different data subsets. The number of genus in full‐length, v3v4, and v5v6 datasets were 44, 60, and 63, respectively. The v3v4/v5v6 sequences included 47 genera, while the full‐length/v3v4 sequences included 39 genera and the full‐length/v5v6 sequences included 39 genera. Thirty‐six genera were present in all datasets. The relative proportion of total sequences assigned to the 36 jointly shared genera was 49.04%, 59.62%, and 43.44% for the full, v3v4 and v5v6 datasets, respectively. In contrast, the proportion of sequences assigned to the 42 remaining ancillary genera ranged between 0.01% and 0.05% (Figure 3b). To further compare taxonomic profiling, the number of sequences assigned to each bacterial taxon was identified (Table 2). This revealed differences in efficiency of read assembly in the different datasets. The discriminant taxon at the order level was Clostridiales with 64.5% and 63.2% higher assignments in the v3v4 and v5v6 data subsets, respectively. Discrepancy is also seen between short sequence length data subsets. An apparent example, which is observed down to the genus level, is sequences assigned to *Carnobacterium* where 23.1% more v3v4 sequences are assigned compared to the full‐length, while the v5v6 data‐subset had only 0.3% assigned compared to the full‐length dataset. The data further suggest differences in the proportion of assigned sequences between sequence length and the taxonomic rank genus and species. Both v3v4 and v5v6 region sequences have a higher proportion assigned to the genus *Flavobacterium* compared to the full‐length dataset. This is opposite to species level where no v3v4 or v5v6 sequences are assigned while 8.3% of the full‐length sequences are assigned to *Flavobacterium frigidarium*.

### 3.3 Bacteria recovered by culture‐dependent methods

**Figure A1:**
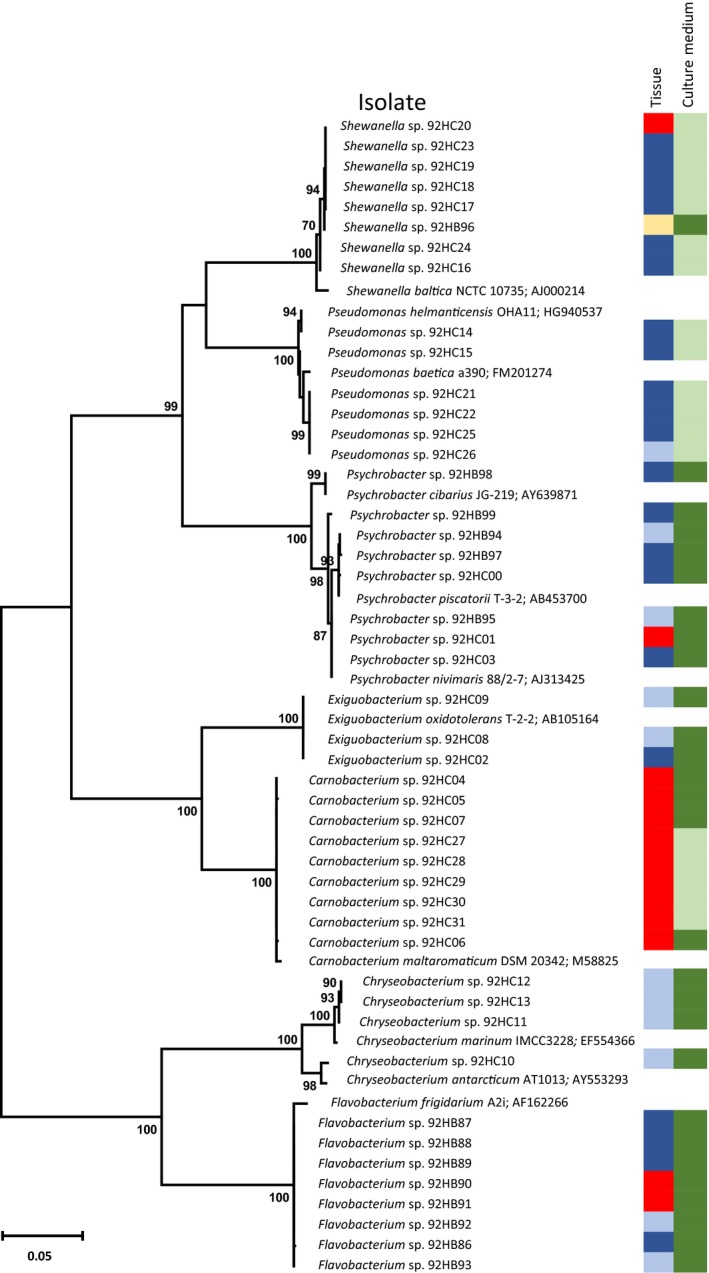
Unrooted neighbor‐joining tree constructed from maximum likelihood distances between 16S rRNA gene sequences obtained from isolates of this study with the most closely aligned type strain obtained from the RDP. Type strains are displayed with strain ID followed by the GenBank 16S rRNA gene accession number. GenBank accession number for isolates obtained in this study is provided in Table A1. Bootstrap values ≥ 70% based on 1,000 replicates are indicated, and the scale bar represents the number of substitutions per site. The right color‐coding columns of the phylogenetic clades show the tissue type each strain was isolated from and culture medium used. Skin is dark blue, gill is light blue, intestine is red, and water is yellow. Culture medium R2A is dark green, and MacConkey is light green

**Figure A2:**
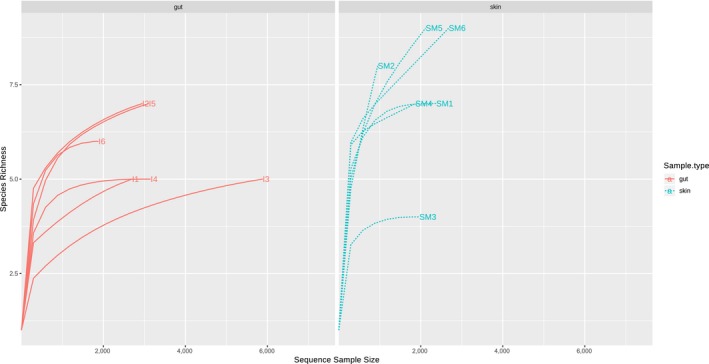
Rarefaction plots based on OTU analysis for intestine and skin samples. Underlaying data represent the number of sequences in each dataset mapped to OTUs clustered with a 97% identity threshold. Plot was generated using total sum scaling of filtered data showing 20 steps

The culture‐based method aimed to provide representative isolates within anticipated genera to compare sequence information against the recovered CCS pool of sequences. Bacterial colonies were phenotypically categorized, and a total of 46 representative colonies were further identified and allocated to seven different genera based on the comparative 16S rRNA gene sequence alignment (Table A1). The relative distribution between colony morphologies on plates retrospectively identified by phylogeny is provided as an average of each sample type in Table A2, Appendix. Isolate information, sample origin, diet group, growth medium used and the most closely related type strains to each genus group is shown in Figure A1, Appendix. Skin mucus samples resulted in *Flavobacterium*, *Psychrobacter,* and *Exigubacterium* isolates on R2A plates, and *Pseudomonas* and *Shewanella* on MacConkey plates. *Carnobacterium* dominated intestinal samples on both media plates. Representative colonies aligning to either genus *Flavobacterium*, *Carnobacterium,* or *Exigubacterium* were identical at the 16S rRNA gene level. Variants within the 16S rRNA gene level were found for isolates within each genus *Chryseobacterium*, *Psychrobacter*, *Pseudomonas,* and *Shewanella*.

### 3.4 Assignment of CCS reads to the bacterial isolates

**Figure 4:**
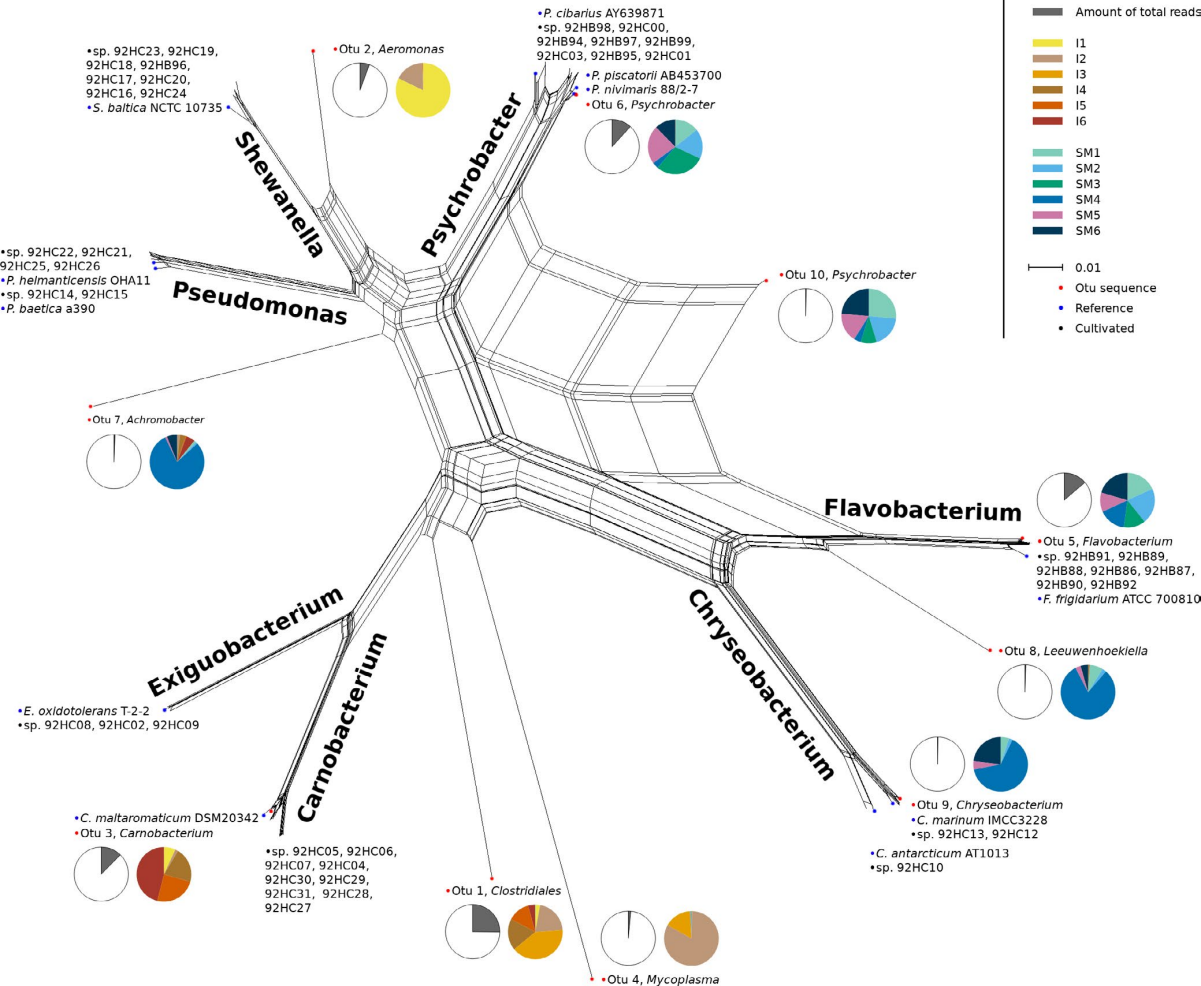
Phylogenetic relationships as NeighborNet network of representative OTUs (red), cultivated bacteria sequences (black), and reference strains (blue). Pie charts detail the given OTU by indicating the proportion of reassigned full‐length 16S reads (gray) and how these are distributed from the samples (colored). The sample values are shown scaled to the minimal sample SM2. Scale bar shows distance as number of nucleotide substitutions per site

The distribution of the 46 identified bacteria isolates among the pool of sequences is shown in Figure 4, where the displayed OTUs in tree branches are representative CCS within each allocated taxon. As expected, not all taxa detected by sequencing are cultivatable under the conditions used. The proportion of cultivable bacteria was investigated by assigning 16S rRNA gene sequences from the obtained isolates to the full‐length 16S rRNA CCS generated reads. Sequences (CCS reads) in cluster with 97% sequence identity to isolates accounted for 3.99% of the CCS reads, that is, cultivability of 3.99%. Comparing the relative abundance of OTUs assigned to the genus level shows domination of *Flavobacterium* and *Psychrobacter* in skin mucus and *Carnobacterium* in the intestinal samples. Two of the most abundant genera in the skin mucus, *Psychrobacter* and *Flavobacterium*, are represented by eight isolates, each (Figure 4). They are clearly distinctive to their respective branch, although the *Psychrobacter* appear to include subgroups that cannot be discriminated based on their 16S rRNA gene sequences (Figure A1). An exception within the tree is created by OTU 10 *Psychrobacter* where the CCS is placed between the genera *Psychrobacter* and *Flavobacterium*. Reads belonging to the genus *Exiguobacterium* was not detected in the intestine and is represented with ≤4 CCS reads in the skin mucus samples. *Shewanella* CCS reads are represented by ≤2 sequences in two samples. No CCS reads belong to the genus *Pseudomonas*.

## 4 DISCUSSION

The skin mucus microbial community was dominated by Proteobacteria within the genus *Flavobacterium* and *Psychrobacter*. This corroborates previous findings reporting the dominance of a few Proteobacteria‐affiliated phylotypes in Atlantic salmon skin mucus from both controlled experiments (Lokesh & Kiron, 2016; Minniti et al., 2017) and commercial production systems (Karlsen et al., 2017). The *Flavobacterium* genus has several important fish pathogens, primarily found in freshwater hatchery‐reared fish (Starliper, 2011), that may adversely affect Atlantic salmon (Loch & Faisal, 2015). *Psychrobacter* spp. are also associated with marine fish species (Ramírez & Romero, 2017a, 2017b; Småge, Frisch, Brevik, Watanabe, & Nylund, 2016). Prominent intestinal bacteria were of the order Clostridiales, Aeromonadales, and Lactobacillales, which are repeatedly reported from the Atlantic salmon intestine (Catalán, Villasante, Wacyk, Ramírez, & Romero, 2017; Gajardo, Jaramillo‐Torres, et al., 2016; Llewellyn et al., 2015; Zarkasi et al., 2014). The number of fish in this trial makes it difficult to provide any conclusive insight into any dietary effect. Of note is the indication of more predominant *Carnobacterium* in the fish fed fish meal. This corroborates a previous study that also substituted fish meal with krill meal followed by culture‐dependent techniques on gut microbiota (Ringø et al., 2006) where *Carnobacterium* was present in non‐krillmeal‐fed fish. A large proportion of the Clostridiales CCS 16S rRNA gene sequences could not be taxonomically assigned above the order rank, indicating the presence of so far uncharacterized bacteria.

Bacteria in the seawater column often inhabit a nutrient poor environment and a large number of the marine bacteria that occur in this environment are suggested to be nonculturable using standard culture‐based techniques (Giovannoni & Stingl, 2005). Bacteria associated with the Atlantic salmon integument are also considered difficult to cultivate. This includes pathogens such as *Tenacibaculum* (Olsen et al., 2011) and *Moritella* (Grove et al., 2008), and culture‐based diagnostics of Atlantic salmon are considered unreliable (Grove et al., 2008). Early studies reported relative high cultivability of bacteria from salmonid intestines (Huber et al., 2004; Spanggaard et al., 2000), but also inconsistencies between culture‐dependent and molecular‐based methods (Hovda, Lunestad, Fontanillas, & Rosnes, 2007). Our approached did not aim to assess culturability, but was applied to recover Atlantic salmon isolates for future studies. Dominant bacteria in the intestine within the order Clostridiales and OTUs assigned to *Aeromonas* and *Mycoplasma* were not isolated, likely due to the growth media and aerobic condition used. Still, an overall ratio of culturability of 3.99% was demonstrated based on the presence/absence of all assigned CCS 16S rRNA gene sequences corresponding to cultivated isolates with 97% sequence identity. This dropped to 0.13% at 99% sequence identity. Both the dominating genera *Flavobacterium* and *Psychrobacter* are studied for their degrading properties (Lasa & Romalde, 2017; Loch & Faisal, 2015), and *Carnobacterium* is isolated from a range of environments (Leisner, Laursen, Prévost, Drider, & Dalgaard, 2007). It is possible that these genera are well‐adapted to grow on the laboratory cultivation media used in this study.

Like many genera associated with the Atlantic salmon, the phylogenetic assignment is often based on the 16S rRNA gene. However, there is a lack of properly defined bacterial species within the aquatic environment that in general hamper our ability to understand and organize bacterial diversity to these environments. There are also technological limitations that may impact on the ecological data description of these analyses (Schmidt, Matias, & Mering, 2015). Related to Atlantic salmon, many of the dominant groups or recovered isolates cannot be discriminated at the species level by their 16S rRNA gene sequences (Grove et al., 2010; Småge et al., 2015). Furthermore, our data suggest that the salmon contains more than one species within observed genera such as *Psychrobacter*. Covering the full‐length sequences of 16S rRNA genes is expected to be advantageous for inferring phylogenetic affiliations and provide a more precise microbial community profiling of Atlantic salmon. However, our partial gene sequences were assigned in a higher abundance compared to the full‐length sequences. Only at the species level was full‐length sequences assigned in a higher proportion (mean 8.9% compared to 0.01% and 0.005% for v3v4 and v5v6 data subsets, respectively). Effects on abundance profiling and discrepancy with an overestimation in bacterial diversity between full‐length 16S rRNA gene and partial variable datasets are in similar accordance with other studies (Singer et al., 2016; Sun et al., 2013; Wagner et al., 2016; Whon et al., 2018). The taxonomic resolution in amplicon sequencing might be affected by several factors. In our study, where we generated different sequence lengths in silico one possibility is differences of the intravariability within each targeted hypervariable region (v3v4 and v5v6) in comparison with the full‐length sequence (Kumar, Brooker, Dowd, & Camerlengo, 2011). Another influencing factor could be the reference sequences in the choice of database used (Werner et al., 2012). Evaluating the species richness, we could not fully describe the community in all samples. The proportion of pooled sequences and the sample sizes obtained can be contributing factors to this lack of convergence where potentially important taxa have not been identified. Using the pooled Atlantic salmon community, we observed discrepancy in community structure and phylogenetic resolution across multiple taxonomic levels between full‐length and partial sequences. Differences were revealed more clearly in some of the phylogenetic lineages. In our data, especially Clostridiales at the order level but also genera within Flavobacteriaceae and Carnobacteriaceae. This highlights that taxonomic affiliation using the bacterial 16S rRNA gene should be concluded with care. The 36 genera shared among the full‐length and partial data subsets compose most of the assigned sequences (49.04%, 59.62%, and 43.44% of the full‐length, and v3v4 and v5v6 data subsets, respectively) and greatly outnumber the proportion of assigned sequences in the 42 ancillary genera identified from one or two of the combined datasets. This high number of genera that is derived from a small part of the sequence sets might indicate that some genera represent false positives, caused by the shorter stretches of the 16S rRNA linked to a lack of resolution in the sequence regions. Collectively, these data suggest that sequence length and part of variable region used on the 16S rRNA gene will influence the outcome of the obtained microbial profile, in a way that is different between taxonomic rank and phylogenetic group. Although this study suggests confounding effects when using different variable regions of the 16S rRNA gene to characterize microbial profiles, it also has key limitations. The sample size should have been increased to better allow robust assessment of experimental effects. The microbiota in context to associations and interactions in animal trials are likely to be complex. Increasing the number of samples may better take in considerations concerning factors such as individual effects caused by husbandry or disease status (Moore & Stanley, 2016). Methodological errors or inadequacy to lyse and extract DNA from bacterial cells between tissue types have also a potential for introducing biases into the results. In addition, the taxonomic resolution in amplicon sequencing might be affected by several additional factors such as PCR conditions used to amplify the product (Lorenz, 2012), primer specificity (Beckers et al., 2016; Tremblay et al., 2015) and possible contamination in low microbial biomass samples (Eisenhofer et al., 2019). To further narrow the gaps and identify the best approach for microbial profiling of Atlantic salmon, future studies should compare primer sets targeting different sequence regions combined with sequencing technology platforms using several tissue types and DNA from a mock community containing a known number of species.

By comparing the distribution of representative OTUs to 16S rRNA gene sequences from cultivated isolates, we aimed to asses any anomaly or difference in the taxonomic classification. The sequences were clearly assigned down to the genus level, except OTU 10 which was placed between the genera *Psychrobacter* and *Flavobacterium*. An interesting observation is that *Exiguobacterium* and *Shewanella* are observed in relative high abundance by the cultivation method compared to the extraction of DNA. *Pseudomonas* was at most represented by five sequences in the pooled v3v4 data subset of the generated 16S rRNA CCS reads. Methodological errors or inadequacy to lyse and extract DNA from bacterial cells, primer specificity, or PCR conditions could be possible explanations. However, because all cultivable taxa were detectable with the nonbarcoded version of the primers it is unlikely to account for an almost complete absence of a genus. Another explanation is that the sequencing depth may have been too low as indicated by the rarefaction analysis. Bacteria not detected by molecular methods may also be site or human‐derived plate contaminants or cultivation procedures may have facilitated growth of these bacteria. The phenomenon of cultivable isolates not being detected in corresponding gene libraries are reported from a wide distribution of marine sample types such as seawater (Eilers, Pernthaler, Glöckner, & Amann, 2000), sponges (Esteves et al., 2016), and algae (KleinJan et al., 2017).

In this study, both skin mucus and intestinal samples were successfully utilized to generate full‐length 16S rRNA gene sequences by the PacBio CCS technology. A high proportion of reads from skin surface samples were allocated down to the level genus. In contrast, intestinal samples dominated by reads assigned to the order Clostridiales were lost at higher resolutions. This highlights the need to further expand the microbial 16S rRNA gene catalogue from underrepresented marine taxa into reference databases. Our data also suggest that different variable regions and sequence length of the 16S rRNA gene will influence the microbial profile differently by taxonomic rank and phylogenetic group. To identify the best taxonomic profiling effectiveness between different variable regions and full‐length 16S rRNA gene would need further validation. However, at present, one of the bottlenecks in comparing microbial profiles is due to the different 16S rRNA gene regions used in different studies. Using the full‐length 16S rRNA gene sequence has the potential to become a tool for more precise microbial community profiling that better allows global comparisons of microbiome studies.

## CONFLICT OF INTERESTS

The authors declare that the research was conducted in the absence of any commercial or financial relationships that could be construed as a potential conflict of interest.

## AUTHOR CONTRIBUTIONS

CK initiated the study and performed the laboratory work. TK conducted and performed the bioinformatic analyses. NW coordinated the work. CK and TK drafted the manuscript and all authors provided critical feedback and helped shape the research, analysis, and manuscript.

## ETHICS STATEMENT

Fish in this study was based on post mortem sampling of material from fish harvested from a different industry research study for other purposes. Fish at the Research Station for Sustainable Aquaculture (Sunndalsøra, Norway) was reared by trained professionals in accordance with guidelines and regulations of the Norwegian Food Safety Authority.

## Data Availability

The datasets generated and analyzed in this study are available in the BioProject repository at https://www.ncbi.nlm.nih.gov/bioproject/PRJEB28410.

## APPENDIX A

**Table A1:** Novel bacterial strains isolated in this study presented with reconstructed phylogenetic genus groups, 16S accession number, isolation source, and culture medium

Isolates	GenBank acc. no.	Tissue	Culture medium
*Flavobacterium* sp. 92HB86	MG263463	Skin	R2A
*Flavobacterium* sp. 92HB87	MG263464	Skin	R2A
*Flavobacterium* sp. 92HB88	MG263465	Skin	R2A
*Flavobacterium* sp. 92HB89	MG263466	Skin	R2A
*Flavobacterium* sp. 92HB90	MG263467	Intestine	R2A
*Flavobacterium* sp. 92HB91	MG263468	Intestine	R2A
*Flavobacterium* sp. 92HB92	MG263469	Gill	R2A
*Flavobacterium* sp. 92HB93	MG263470	Gill	R2A
*Psychrobacter* sp. 92HB94	MG263471	Gill	R2A
*Psychrobacter* sp. 92HB95	MG263472	Gill	R2A
*Psychrobacter* sp. 92HB97	MG263473	Skin	R2A
*Psychrobacter* sp. 92HB98	MG263474	Skin	R2A
*Psychrobacter* sp. 92HB99	MG263475	Skin	R2A
*Psychrobacter* sp. 92HC00	MG263476	Skin	R2A
*Psychrobacter* sp. 92HC01	MG263477	Intestine	R2A
*Psychrobacter* sp. 92HC03	MG263478	Skin	R2A
*Exiguobacterium* sp. 92HC02	MG263480	Skin	R2A
*Exiguobacterium* sp. 92HC08	MG263481	Gill	R2A
*Exiguobacterium* sp. 92HC09	MG263482	Gill	R2A
*Chryseobacterium* sp. 92HC10	MG263487	Gill	R2A
*Chryseobacterium* sp. 92HC11	MG263488	Gill	R2A
*Chryseobacterium* sp. 92HC12	MG263489	Gill	R2A
*Chryseobacterium* sp. 92HC13	MG263490	Gill	R2A
*Pseudomonas* sp. 92HC14	MG263491	Skin	MacConkey
*Pseudomonas* sp. 92HC15	MG263492	Skin	MacConkey
*Pseudomonas* sp. 92HC21	MG263493	Skin	MacConkey
*Pseudomonas* sp. 92HC22	MG263494	Skin	MacConkey
*Pseudomonas* sp. 92HC25	MG263495	Skin	MacConkey
*Pseudomonas* sp. 92HC26	MG263496	Gill	MacConkey
*Shewanella* sp. 92HB96	MG263479	Water	R2A
*Shewanella* sp. 92HC16	MG263497	Skin	MacConkey
*Shewanella* sp. 92HC24	MG263498	Skin	MacConkey
*Shewanella* sp. 92HC17	MG263499	Skin	MacConkey
*Shewanella* sp. 92HC18	MG263500	Skin	MacConkey
*Shewanella* sp. 92HC19	MG263501	Skin	MacConkey
*Shewanella* sp. 92HC20	MG263502	Intestine	MacConkey
*Shewanella* sp. 92HC23	MG263503	Skin	MacConkey
*Carnobacterium* sp. 92HC27	MG263504	Intestine	MacConkey
*Carnobacterium* sp. 92HC28	MG263505	Intestine	MacConkey
*Carnobacterium* sp. 92HC29	MG263506	Intestine	MacConkey
*Carnobacterium* sp. 92HC30	MG263507	Intestine	MacConkey
*Carnobacterium* sp. 92HC31	MG263508	Intestine	MacConkey
*Carnobacterium* sp. 92HC04	MG263483	Intestine	R2A
*Carnobacterium* sp. 92HC05	MG263484	Intestine	R2A
*Carnobacterium* sp. 92HC06	MG263485	Intestine	R2A
*Carnobacterium* sp. 92HC07	MG263486	Intestine	R2A

**Table A2:** Overview of the most abundant bacteria assigned to the genus level present in Atlantic salmon sampled skin mucus and intestine. Their relative abundance across sample types are obtained either from sequence‐based profiles (CCS reads) or from culture depended method assessing colony morphologies. Values are relative abundance in percentage, mean ± *SD*

Tissue	Extracted DNA				Bacteria on culture medium							
	Skin		Intestine		Skin				Intestine			
Diet	Fish meal	Krill meal	Fish meal	Krill meal	Fish meal		Krill meal		Fish meal		Krill meal	
Growth medium					R2A	MC	R2A	MC	R2A	MC	R2A	MC
*Flavobacterium*	50.5 ± 16.1	46.7 ± 12.3	0.9 ± 0.3	1.0 ± 1.1	54.5 ± 29.9		82.3 ± 8.1		0.5 ± 0.6			
*Psychrobacter*	34.1 ± 20.6	49.6 ± 14.7	0.7 ± 0.4	0.5 ± 0.5	45.5 ± 29.8		17.0 ± 7.0		0.3 ± 0.6			
*Carnobacterium*	0.3 ± 0.1	0.2 ± 0.2	97.4 ± 0.5	11.0 ± 8.3					99.1 ± 0.3	99.5 ± 0.7	100	100
*Aeromonas*	0.1 ± 0.2	0.1 ± 0.1	0.3 ± 0.3	44.0 ± 38.9								
*Chryseobacterium*	8.7 ± 0.7	2.4 ± 1.9	0.2 ± 0.1	0.1 ± 0.2								
*Mycoplasma*	0.0 ± 0.0	0.0 ± 0.0	0.1 ± 0.1	42.9 ± 45.1								
*Leeuwenhoekiella*	4.0 ± 6.2	0.4 ± 0.5	0.1 ± 0.1									
*Pseudoalteromonas*	1.2 ± 0.6	0.3 ± 0.3										
*Pseudomonas*						2.4 ± 2.1		28.0 ± 11.3				
*Shewanella*						97.6 ± 2.1		72.0 ± 11.3		0.5 ± 0.7		
*Exiguobacterium*	0.1 ± 0.0						0.8 ± 1.1					
